# The Relationship Between Diabetes Mellitus and Cancers and Its Underlying Mechanisms

**DOI:** 10.3389/fendo.2022.800995

**Published:** 2022-02-11

**Authors:** Bing Zhu, Shen Qu

**Affiliations:** Department of Endocrinology and Metabolism, Shanghai Tenth People’s Hospital, School of Medicine, Tongji University, Shanghai, China

**Keywords:** cancers, mechanism, type 2 diabetes (T2D), type 1 diabetes, type 3C diabetes mellitus

## Abstract

Epidemiological studies suggest associations between diabetes mellitus and some cancers. The risk of a number of cancers appears to be increased in diabetes mellitus. On the other hand, some cancer and cancer therapies could lead to diabetes mellitus. Genetic factors, obesity, inflammation, oxidative stress, hyperglycemia, hyperinsulinemia, cancer therapies, insulin and some oral hypoglycemic drugs appear to play a role in the crosstalk between diabetes mellitus and cancers. This review summarized the associations between various types of diabetes and cancers and updated available evidence of underlying mechanisms between diabetes and cancers.

## Introduction

The link between diabetes and cancer has been proposed for more than 100 years (1). The risk of cancers appears to be increased in both type 1 diabetes mellitus (T1DM) and type 2 diabetes mellitus (T2DM) (2). Cancer was also reported to be the second most common cause of death for people with T1DM (3). On the other hand, approximately 8%-18% of patients with cancer have diabetes (4). Further, previous studies have suggested that diabetes is associated with increased risk of cancer mortality (5, 6). However, the underlying mechanisms between various types of diabetes and cancers have not yet been summarized. This review summarizes the associations between various types of diabetes and cancers, and updated available evidence underlying mechanisms between diabetes and cancers.

## Incidence and Mortality of Cancers in Patients With Diabetes Mellitus

### T1DM

A five-country study of cancers in patients with T1DM has reported that T1DM was correlated with the risk of several common cancers. For non-sex-specific cancers, the estimated hazard ratio (HR) and 95% confidence intervals (CIs) for overall cancer were 1.15 (1.11, 1.19) among men and 1.17 (1.13, 1.22) among women when compared to the general population (7). Cancer incidence of liver, pancreas, kidney, esophagus, stomach, lung, thyroid, squamous cell carcinoma, and leukaemia significantly increased for both sexes with T1DM (7–10). Incidence of non-Hodgkin’s lymphoma and colon cancer significantly increased for men (7); while incidence of the ovary, esophagus, endometrium, vulva and vagina, and thyroid cancer significantly increased for women (7, 11, 12). On the contrary, incidences of prostate cancer and testis cancer significantly decreased in men with T1DM in comparison with the general population (7, 13). Women with T1DM had significantly lower risk for breast cancer, melanoma, and Hodgkin’s lymphoma (7, 10). Previous cohort studies also reported an overall increased standardized mortality ratio for cancers among patients with T1DM compared with the general population (14).

Several studies generated inconsistent findings. Some early studies found no significant associations between T1DM and a range of site-specific cancers (15). Previous large cohort studies conducted in the UK suggested that neither the risk of urinary bladder cancer nor mortality from urinary bladder cancer was increased in patients with T1DM or T2DM (11, 16). This is in line with the results found in other study undertaken in Sweden (9, 17). Similarly, some studies found no significant association between the risk of breast cancer and T1DM in women (11, 17). In addition, cohort studies undertaken in the UK (11, 18) and the USA (19) reported that no significantly increased all-cause cancer mortality was found in patients with T1DM when compared to the general population. But, there was evidence of heterogeneity in risk of some cancers by country, and T1DM duration (7). Thus, study population selection (e.g., ethnicity, age range, and gender), study design, publication bias, other demographic and socioeconomic factors should be considered when interpreting these results.

### T2DM

A comprehensive meta-analysis has concluded that the presence of T2DM is associated with approximately 10% increase of the risk to develop cancer (5). Previous studies have provided substantial evidence of associations between T2DM and risks of cancer in hepatocellular, biliary tract, gallbladder, pancreas, gastrointestinal, kidney, bladder, lung, thyroid, breast, ovarian, endometrial, oral, leukemia, glioma, and melanoma (5, 20–25). Among them, the highest risks has been demonstrated for colorectal cancer (26), hepatocellular cancer (27), or pancreatic cancer (28).

On the contrary, some cancers showed a null or decreased risk in diabetic patients in some studies, including brain, buccal cavity, esophageal, lung, breast, urinary bladder, and laryngeal cancer (20). It is worth noting that numerous studies that were conducted in Americans and Europeans indicated a reduced risk of prostate cancer in patients with T2DM (29, 30). Moreover, the protective effect was more evident for patients with more than 10 years T2DM duration (13). Indeed, men with diabetes had lower levels of testosterone (31) than those without, and testosterone has been demonstrated to be associated with an elevated risk of prostate cancer (32). Additionally, studies with genome-wide association analyses indicated that HNF1B gene variants would not only drive haplotype-carrying people to diabetes, but also protect them from prostate cancer (33). However, studies in Asians reported contradicting results, and large meta-analyses suggested that there was a positive association between T2DM and prostate cancer in Asians (30, 34).

Previous large meta-analyses have estimated that diabetes is associated with 25%-41% increased risk of mortality from any cancer (35, 36). In a prospective cohort conducted in US adults, diabetes was related to increases in any cancer mortality of 7% in men and 11% in women, respectively (29). In an analysis of 19 Asian cohorts followed for up to 21 years, T2DM was related to a 26% increase in the risk of cancer mortality (6). Significant positive associations between T2DM and mortality from cancers were observed for the cancers of stomach, colorectum, oral cavity, gallbladder, bile duct, liver, pancreas, ovary, endometrium, breast, thyroid, prostate, lung, kidney, bladder, and lymphoma (6, 22, 37). Controversially, some studies reported a null association between T2DM and the risk of death from cancers of the lung, bladder, stomach, cervix, esophagus, as well as leukaemia (6, 38), suggesting that the role of diabetes in these site cancer needs further clarification.

### Type 3c Diabetes (T3cDM) or Pancreoprivic Diabetes

Type 3c diabetes (T3cDM) or pancreoprivic diabetes is caused by various diseases of the exocrine pancreas (39). The diverse causes of T3cDM include pancreatic carcinoma, acute and chronic pancreatitis, cystic fibrosis, trauma or pancreatectomy, fibrocalculous pancreatopathy, hemochromatosis, idiopathic forms, and rare genetic disorders. Pendharkar et al. showed that the prevalence of diabetes in individuals with exocrine pancreas diseases was approximately 0.11% (40). Ewald N et al. reported that approximately 9.2% of patients with diabetes were identified as T3cDM (41).

A comprehensive meta-analysis showed that the relative risk of pancreatic cancer was negatively associated with the diabetes duration, with the highest risk of pancreatic cancer found among patients whose diabetic history within less than 1 year (42). It indicates that diabetes may have resulted from undiagnosed pancreatic cancer (43). Indeed, T3cDM occurs in up to 30% of patients with pancreatic cancer (44). On the other hand, successful treatment of pancreatic cancer could improve hyperglycemia for patients with T3cDM due to pancreatic cancer (45). Additionally, the risk of pancreatic cancer has been increased 10- to 20-fold in patients with chronic pancreatitis, which is the most common cause of T3cDM; this risk has been increased 33-fold in patients with the combination of chronic pancreatitis and diabetes mellitus (46). A previous study estimated that approximately 10% T3cDM patients had pancreatic cancer (41).

Animal studies found the presence of hyperinsulinemia (47) and insulin secretory impairments (48) in pancreatic cancer models. Indeed, euglycemic glucose clamp studies demonstrated that both the insulin sensitivity and beta-cell function were markedly impaired in patients with pancreatic cancer (45, 49). T3cDM secondary to pancreatic cancer seems to be related to the mediators released by cancer (50). Adrenomedullin was identified as one of the key mediators for beta-cell toxicity in a cell-line study of pancreatic cancer (51). Further, a clinical study reported that the levels of adrenomedullin are higher in patients with pancreatic cancer-induced diabetes in comparison to general population (52). In addition, the upregulation of connexin and S100A8/A9 in pancreatic tissues could attenuate the glucose utilization (53, 54). Furthermore, interleukin-1β and tumor necrosis factor (TNF)-α are found abundant in a tumor microenvironment in diabetes due to pancreatic cancer (55), which somehow explains the impaired beta-cell function observed in patients with pancreatic cancer (56).

## Cancer Treatment and Diabetes

### Chemotherapy

Most chemotherapeutic agents result in the cell cycle or cellular DNA damage and thus leading to apoptosis disproportionately in rapidly dividing cells. A number of studies reported that patients who received chemotherapy such as Tegafur-uracil (UFT) (57), paclitaxel (58), or interferon alpha (59) had developed fulminant T1DM or autoimmune-mediated T1DM. Mouse studies indicated that interferon alpha causes autoimmune diabetes by promoting the maturation of conventional dendritic cells and the activation of B cells. Further, interferon alpha could directly damage pancreatic beta cell functions by inducing cytokines and enhancing their susceptibility to invasion by diabetogenic T cells (60). Diabetes is also a rare complication of UFT use. UFT could cause fulminant T1DM through immune suppression or an immunological reaction, and the effects of thymidine phosphorylase (57).

### Glucocorticoid

Glucocorticoids are a commonly used treatment for cancers of blood system (61). Additionally, they are used to treat cancer pain, chemotherapy-induced side-effects such as nausea and vomiting, and cancer-related cachexia (62, 63). Furthermore, they have an ancillary role in treatment of inflammatory complications of cancer therapy and autoimmune conditions of immunomodulatory therapies (64). Steroid-induced diabetes mellitus has been recognized as a complication of glucocorticoid use for over 50 years (65). A previous study found that patients with previously well controlled T1DM treatment with 60 mg prednisone daily for 3 days led to deterioration of glycemic control despite average 70% increase of insulin dosage (66). It is likely that glucocorticoid administration causes hyperglycemic states or diabetes mellitus by impairing pancreatic beta-cell functions and insulin sensitivity (67). An *in vitro* study observed impaired insulin secretion of prednisone-treated INS-1E cells in response to a glucose challenge. On the contrary, this phenomenon was reversed in the presence of prednisone with the glucocorticoid receptor antagonist, RU486 (68). Glucocorticoids could induce insulin resistance through several mechanisms. For example, glucocorticoids increase the levels of serum fatty acids by regulating the expression of PEPCK gene in adipose tissue and liver and controlling glyceroneogenesis. It is well known that an increase in fatty acids interferes with glucose utilization and results in insulin resistance (69). Moreover, glucocorticoids decrease insulin sensitivity by directly interfering with components of the insulin signaling cascade, such as glycogen synthase kinase-3, glycogen synthase and GLUT4 translocation (67, 70).

### Targeted Cancer Therapies

Targeted cancer therapies attempt to treat cancer by targeting the changed cellular pathways that drive unregulated growth. This treatment can somehow impair insulin sensitivity since some altered cellular pathways are linked to the actions of insulin. For instance, the anti–insulin like growth factor 1 receptor (IGF-IR) inhibition has been long proposed as a treatment strategy of various cancers (71, 72). A phase I dose escalation study of the Anti-IGF-IR monoclonal antibody CP-751,871 in patients with refractory solid tumors reported that the treatment with CP-751,871 increased serum glucose levels (73). It is likely that the levels of growth hormone (GH) increase after IGF-1 blockade, thereby leading to an increase in insulin resistance (74). In addition, mammalian target of rapamycin (mTOR) inhibitors have been used for multiple types of cancer such as breast cancer and renal cell carcinoma. Data from clinical trials suggested that a treatment with mTOR inhibitors was associated with a high incidence of hyperglycemia and new-onset diabetes, ranging from 13% to 50% (75). The mechanisms responsible for hyperglycemia with mTOR inhibitors are likely due to the combination of impaired insulin secretion and insulin resistance (76, 77). Hyperinsulinemia and hyperglycemia were also seen after administrations target the proteins in the same pathway, including PI3 kinase and Akt in mice (78).

### Cancer Immunotherapy

Cancer immunotherapies, including immune checkpoint inhibitors, adoptive cell therapy, oncolytic viruses, and cancer vaccines, manipulate the immune system to recognize and attack cancer cells. These therapies have the potential to lead to toxicity profiles for endocrine system. For instance, insulin-dependent diabetes has been reported in patients treated with anti-programmed cell death protein 1 (PD-1) or anti-programmed cell death ligand-1 (PDL-1) antibodies (79). The prevalence of diabetes was estimated at 0.4%-0.9% in this population (80–82). Animal studies also indicated that anti-PD-1 or anti-PDL-1 antibody injection triggered onset of diabetes in mice (83, 84). But, to date, the exact mechanism is poorly known. Histologic analysis of the pancreas found massive destructive insulitis in mice receiving anti-PD-1 or anti-PD-L1 antibodies.

## Mechanisms Underlying the Association Between Diabetes Mellitus and Cancers

### Genetic Background

Genetic factors have been identified as contributing to the associations between diabetes and some cancers. For instance, individuals who have a family history of pancreatic cancer often have a higher risk of developing pancreatic cancer (85). Indeed, several studies reported that the glucose-raising allele of MADD rs11039149, MTNR1B rs1387153, FTO rs8050136 per allele, glucokinase regulator rs780094 of T2DM were positively associated with the risk of pancreatic cancer (86, 87).

### Common Risk Factors

#### Obesity

It is well known that most patients with prediabetes or T2DM have overweight or obesity (39). A large cohort study which included 900,000 individuals with an over 16-year duration of follow-up reported that severe obesity was associated with a significantly increased mortality from cancers of the liver, pancreas, colon and rectum, kidney, non-Hodgkins lymphoma, esophagus, and multiple myeloma. The greatest influences were observed in cancers of liver, colon and rectum, and pancreas (88). Additionally, a lower incidence of obesity-related cancers (89) and a significant reduction of cancer-related medical care (90) were found in bariatric surgery patients when compared with morbidly obese controls.

Obesity may act as an important confounder or an effect modifier in the relationship between T2DM and cancer (4). Obesity was associated with increased risk of cancers probably by mechanisms that involve cellular proliferation, inflammation, and hormonal balance (91), which have also been supposed for the relationship between T2DM and cancer. Taking pancreas for example, Butler et al. studied the effects of obesity and diabetes mellitus on pancreatic ductal pathology and found that the replication of pancreatic duct was increased ten-fold in specimens obtained from obese nondiabetics compared with lean nondiabetics, and duct epithelial replication was increased four-fold in lean diabetics in comparison with lean nondiabetics. These results suggest the independent effects of diabetes and obesity on the risk of the development of pancreatic exocrine neoplasia (92).

#### Inflammation and Oxidative Stress

Inflammation is a key element in the link between diabetes mellitus and cancer (93). T2DM is associated with insulin secretory defects related to inflammation (39). Chronic inflammation, which is characterized by high levels of oxidative stress and reactive oxygen species (ROS), activation of pro-inflammatory pathways, and abnormal adipokine production, may establish a micro-environment thereby promote tumor cell growth, enhance metastasis, increase angiogenesis and impair the function of natural killer cells and macrophages (94).

Oxidative stress plays an important role in the crosstalk between cancer and diabetes. Hyperglycemia could increase superoxide production (95). Furthermore, insulin could stimulate reactive oxygen species (ROS) production (96). It has been confirmed that oxidative stress has a strong influence on a number of genes expression and signal transduction pathways that have an important role in tumorigenesis (97). ROS have been demonstrated to interfere with cell proliferation and apoptosis by activating cytokine-dependent activation of nuclear factor (NF)-кB pathways (98). NF-кB was demonstrated to be hyperactivated in colorectal cancer (99), breast, blood neoplasms, and pancreas cell lines (97, 100).

### Hyperglycemia

Epidemiological data have shown that hyperglycemia is related to higher risk of colorectal, liver, gastric, lung and pancreatic cancer (101, 102). The phenomenon termed “the Warburg effect” partly explains why hyperglycemia favors tumorigenesis (103). Normally, cells differentiates rely on mitochondrial oxidative phosphorylation to provide the energy to cellular processes, while cancer cells tend to use a less efficient glycolytic pathway for proliferation (103, 104). Cancer cells therefore require increased glucose uptake to generate sufficient energy hence meet their proliferation needs (105). The cancer predisposition associated with diabetes may result from imbalance of signal transduction pathways that manage the utilization of nutrient and fuels (106).

Hyperglycemia stimulates the production of advanced glycation end products (AGEs). AGEs often interact with their specific receptor, RAGE, activate NF-кB and generate ROS in cells, thereby accelerating oxidative stress that leads to increased proinflammatory signaling (107). Activation of the AGEs pathway has been demonstrated to promote tumor transformation of epithelial cells (108). Clinical tests also confirmed a positive association between the AGE/RAGE interaction and risk of gastric cancer (109), pancreatic cancer (110), and melanoma (111). In addition, Han et al. reported that hyperglycemia stimulates proliferation of pancreatic cancer cell *via* the induction of epithelial growth factor (EGF) expression and transactivation of the EGF receptor (112). Furthermore, hyperglycemia has been supposed to damage the lung structure, which is the basis for lung cancer (113). Moreover, hyperglycemia is responsible for DNA damage, which is the first stage of tumorigenesis (114).

### Hyperinsulinemia

Several epidemiological studies have shown that hyperinsulinemia is associated with an increased risk for several cancers, including cancers of the endometrium, ovarian, breast, colon, pancreas, and kidney (115, 116). Indeed, both *in vitro* and *in vivo* studies demonstrated that insulin and insulin receptor (IR) played a key role in cancer biology (117). In hyperinsulinemic states, the hepatic IGF-1 production increased due to the upregulation of the growth hormone receptor (GHR) and augment of GHR signaling (118). Epidemiological studies and meta-analyses suggested that higher IGF-1 levels were correlated with an increased risk of colorectal, lung, premenopausal breast and prostate cancer (119). Animal studies confirmed that IGF-1 administration increased the cancer cells proliferation and their capacity to spread in secondary sites. On the contrary, knock-out of the *Igf-1* gene inhibited growth of the tumor (120). In addition, IGF-2 overexpression has been also associated with colon cancer development in mouse models (121). Insulin, IGF-1 and IGF-2 could activate the PI3K/Akt/mammalian target of rapamycin (mTOR) signaling pathway, thereby promoting the development of cancers (122).

### Exogenous Insulin and Insulin Analog Therapy

There is some evidence that patients with insulin therapy have a higher incidence of cancers when compared to patients with no insulin use (123), including cancers in colorectum, breast, pancreas, liver, kidney, stomach and respiratory system (124, 125). A retrospective study showed that patients treated with insulin agents such as human insulin, aspart, lispro and glargine exhibited a dose-dependent increased risk of cancer development (126). An animal study showed that insulin administration increased colonic epithelial tissue proliferation, thereby promoted colon cancer growth (127). The possible mutagenic effects of insulin or insulin analog and increased levels of IGF-1 might be the potential biological plausibility for the increase risks of cancers (128, 129). It should be kept in mind that insulin analogs may have a metabolic action and a mitogenic action altered from that of human insulin (130). Further, compared to insulin, the mitogenic pathways may be more activated when using long-acting analogues (131). However, some previous large randomized controlled trial study (132), cohort study (133), and systematic review (134) concluded that insulin (analog) treatment does not impact the risk of cancer overall and some site-specific cancers.

### Oral Hypoglycemic Drugs

#### Metformin

Numerous clinical studies and meta-analyses have demonstrated that diabetes exposure to metformin was associated with a significantly decreased cancer incidence and mortality (135–137). Moreover, the addition of metformin ameliorates the increased risk of cancer in patient therapy with sulfonylurea or insulin (138). Studies in animal models and in cancer cell lines *in vitro* complemented these results that metformin could inhibit development of cancer (139). The potential mechanism is that metformin may inhibits the mTOR in an adenosine monophosphate (AMP)-activated protein kinase (AMPK)-dependent manner, concomitant reduces insulin levels, and increases insulin sensitivity (139, 140). Metformin could also inhibit tumorigenesis by modulating several other targets such as STAT3, TP, p53, etc. (140). In addition, metformin has been demonstrated to enhance the activity of several cancer drugs such as platinum compounds (140). Recently, the METAL (METformin in Advanced Lung cancer) study provided evidence that metformin plus erlotinib in second-line treatment of patients with stage IV NSCLC prolonged median progression-free survival (141). Moreover, Morgillo et al. demonstrated that metformin increases the antitumor activity of MEK inhibitors in human LKB1-wild-type non-small cell lung cancer cell (NSCLC) lines by reducing the NF-kB (p65)-mediated transcription of MMP-2 and MMP-9 and through downregulation of GLI1 (142).

#### Glucagon-Like Peptide-1 Receptor Agonist and Dipeptidyl Peptidase-IV Inhibitor

Incretin-based therapy, including dipeptidyl peptidase-IV (DPP-IV) inhibitor and glucagon-like peptide-1(GLP-1) receptor agonist is increasingly used in T2DM. Elashoff et al. reported that the use of DDP-IV inhibitor sitagliptin or the GLP1 analog exenatide was associated with a significant increase in the incidence of pancreatic cancer (143). Indeed, Matveyenko et al. observed that sitagliptin induced replication and apoptosis of beta-cell, pancreatic ductal metaplasia, and a four-fold increase in duct cell proliferation, suggesting that sitagliptin is the risk factor for the development of pancreatic cancer (144). Furthermore, animal studies showed that exendin-4, the GLP-1 analog, increased duct cell replication and the development of dysplastic pancreatic intraepithelial neoplasia lesions (145). In addition, liraglutide, a GLP-1 receptor agonist, was associated with increased risk of thyroid c-cell focal hyperplasia, indicating an increased risk of medullary cell thyroid cancer (146). However, a meta-analysis suggested that there is no exact evidence that the risk of pancreatic cancer in patients on incretin-based therapies is significantly higher than that in patients on other therapies (147, 148).

#### Sodium-Glucose Linked Transporter 2 (SGLT 2) Inhibitors

A meta-analysis suggested that the risk of bladder cancer might be increased in patients with SGLT2 inhibitors, especially with empagliflozin (149). However, this association was not confirmed by other authors (150, 151). Scafoglio C et al. even suggested that SGLT2 inhibitors may be useful for cancer therapy (152), as SGLT2 inhibitor was associated to increased tumor necrosis and hence induced tumor shrinkage (152). Indeed, canagliflozin was demonstrated to inhibit cancer growth by inhibiting the complex I of the mitochondrial respiratory chain (153).

#### Sulfonylureas (SUs)

Previous studies have indicated that patients treated with sulfonylureas therapy have an increased incidence of cancer and risk of cancer mortality (154, 155), particularly in pancreatic (138) and breast cancer (156). However, some randomized controlled trials showed no statistically significant difference in the risk of cancer between the use of SUs and other treatments (157).

#### Thiazolidinediones (TZDs)

TZDs have potent insulin-sensitizing activity used to improve lipid and glucose metabolism through the activation of peroxisome proliferator-activated receptors (PPARs) (158). In 2005, the PROactive Study firstly proposed the positive association of bladder cancer with pioglitazone use in patients with T2DM (159). However, pioglitazone bladder cancer concerns have been largely attenuated by recent evidence (160). Lv et al. demonstrated that the activation of PPARγ induced cell cycle G2 arrest and inhibition of bladder cancer cells proliferation by inhibiting the PI3K-Akt pathway *in vitro* (161). Additionally, PPAR-γ activation has been found to inhibit the growth of other tumor cells such as colon, breast and lung cancer cell lines through induction of apoptosis (162, 163). Ciaramella et al. investigated the anti-tumor effects of pioglitazone in NSCLC cell lines and found that pioglitazone reduced proliferative and invasive abilities and induced apoptosis of NSCLC cells by inhibiting MAPK/AKT cascade as well as on the TGFβ/SMADs system (164). Indeed, Mazzone et al. indicated that the TZDs treatment was associated with a lower risk of developing lung cancer in patients with diabetes (165). A meta-analysis also suggested that TZDs were associated with a significantly lower risk of colorectal and breast cancer (166, 167). In addition to anti-proliferative effects, TZDs can also enhance cytotoxic effects of some anticancer therapies such as cisplatin and oxaliplatin by increasing the expression of apoptosis-inducing factor (AIF) and suppressing survivin (168).

## Conclusion

**Figure 1:**
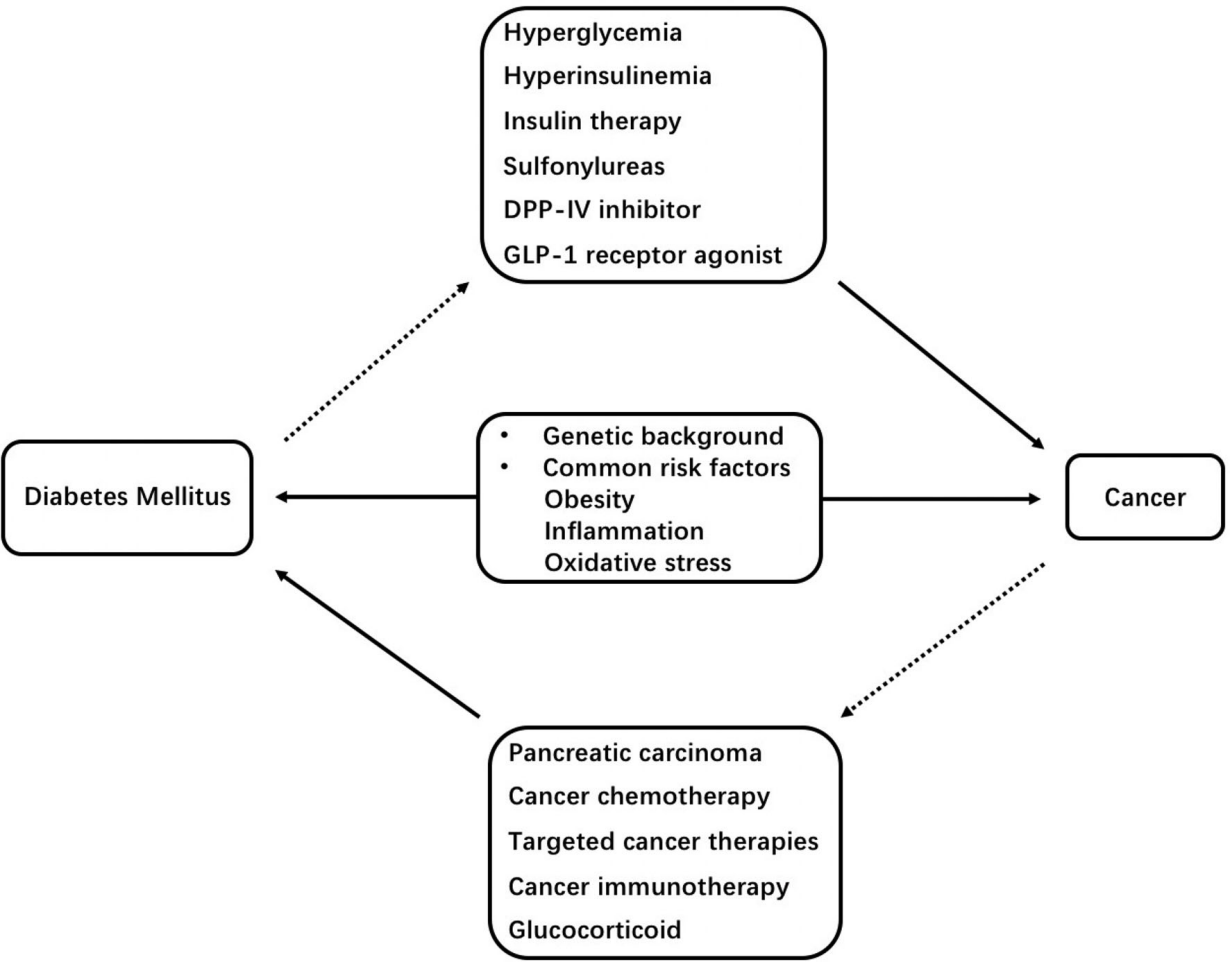
Schematic representation of mechanisms underlying the association between diabetes mellitus and cancers. DPP-IV, dipeptidyl peptidase-IV; GLP-1, glucagon-like peptide-1.

There is a complicated association between diabetes mellitus and cancers. In summary, the risk of a number of cancers and cancer mortality is increased in T1DM and T2DM. On the other hand, some kinds of cancer and cancer therapies are associated with the increased risk of diabetes mellitus. Additionally, genetic factors, obesity, inflammation, oxidative stress, hyperglycemia, hyperinsulinemia, cancer therapies, insulin and some oral hypoglycemic drugs appear to play a role in the crosstalk between diabetes mellitus and cancers (**Figure 1**). Thus, we suggest that cancer screening should be conducted in patients with diabetes, and precautions for diabetes should be taken in patients suffering from cancer. Further researches are merited to explore on the associations between these different diseases.

## Author Contributions

Both authors have met the requirements for authorship. BZ and SQ summarized and edited the manuscript. Both authors have read and approved the final manuscript.

## Funding

This work was supported by the National Key R&D Program of China (No.2018YFC1314100, SQ), the National Natural Science Foundation of China (82100903, BZ), and the Shanghai Sailing Program (21YF1435200, BZ).

## Conflict of Interest

The authors declare that the research was conducted in the absence of any commercial or financial relationships that could be construed as a potential conflict of interest.

## Publisher’s Note

All claims expressed in this article are solely those of the authors and do not necessarily represent those of their affiliated organizations, or those of the publisher, the editors and the reviewers. Any product that may be evaluated in this article, or claim that may be made by its manufacturer, is not guaranteed or endorsed by the publisher.
